# Tamoxifen induces hypercoagulation and alterations in ERα and ERβ dependent on breast cancer sub-phenotype ex vivo

**DOI:** 10.1038/s41598-020-75779-y

**Published:** 2020-11-06

**Authors:** K. Pather, T. N. Augustine

**Affiliations:** School of Anatomical Sciences, Faculty of Health Sciences, University of the Witwatersrand, 7 York Road, Parktown, Johannesburg, 2193 South Africa

**Keywords:** Breast cancer, Platelets

## Abstract

Tamoxifen shows efficacy in reducing breast cancer-related mortality but clinically, is associated with increased risk for thromboembolic events. We aimed to determine whether breast tumour sub-phenotype could predict propensity for thrombosis. We present two ex vivo Models of Tamoxifen-therapy, Model 1 in which treatment recapitulates accumulation within breast tissue, by treating MCF7 and T47D cells directly prior to exposure to blood constituents; and Model 2 in which we recreate circulating Tamoxifen by treating blood constituents prior to exposure to cancer cells. Blood constituents included whole blood, platelet-rich plasma and platelet-poor plasma. Hypercoagulation was assessed as a function of thrombin activity, expression of CD62P and CD63 activation markers defined as an index of platelet activation, and platelet morphology; while oestrogen receptor expression was assessed using immunocytochemistry with quantitative analysis. We determined, in concert with clinical studies and contrary to selected laboratory investigations, that Tamoxifen induces hypercoagulation, dependent on sub-phenotypes, with the T47D cell line capacity most enhanced. We determined a weak positive correlation between oestrogen receptor expression, and CD62P and CD63; indicating an association between tumour invasion profiles and hypercoagulation, however, other yet unknown factors may play a predictive role in defining hypercoagulation.

## Introduction

Breast cancer is one of the most commonly diagnosed cancers, accounting for 24.2% of 8.6 million new cases in women worldwide^1,2^. Targeted treatment strategies, including Tamoxifen, a selective oestrogen receptor modulator (SERM) for hormone-dependent breast tumours, show efficacy in reducing mortality and increasing survival^3,4^. Tamoxifen can be taken orally or intravenously, depending on the severity, staging and patient preference^5^, and is known to increase a patient’s risk for thrombosis^6,7^. Tamoxifen used as an adjuvant treatment strategy for breast cancers in post-menopausal women, indicated positive disease-free state with an associated greater risk for development of venous thromboembolisms (VTE)^7–9^. While Tamoxifen has shown efficacy in reducing mortality and increasing survival in patients^1–4,10–13^, associated and resulting thrombosis remains the second main cause of mortality^1,8–11,14^. This induction of hypercoagulation indicates an interplay between oestrogen-induced platelet activation as well as activation due to the cytotoxic nature of Tamoxifen itself^15–17^. The precise mechanism by which Tamoxifen exerts its procoagulant effect remains to be discovered^7,18^; however, studies implicate the anti-oestrogenic activity of the drug on oestrogen receptor alpha (ERα), particularly on those expressed by platelets , to further increase the risk of VTE^19,20^; and further effects on Factor VIII in promoting thrombotic events via its role in platelet activation^18^.

Platelets are key players in hypercoagulation and their role in tumour progression is becoming increasingly evident. Tumour cells facilitate platelet activation and aggregation, and the formation of a fibrin-rich network, by either direct contact with platelets or by the release of agonists including tissue factor, ADP, thrombin or thromboxane A2 (TXA2)^21,22^. Of these agonists, thrombin is proposed to be the most potent contributor to platelet activation acting via engagement of platelet protease-activated receptors (PAR1-4) and ultimately inducing the coagulation cascade^23–25^. In turn, intratumoral platelets facilitate the acquisition of a more invasive phenotype in preparation for tumour cell intravasation and subsequent metastasis^26–28^. In the circulation, platelets form heterotypic aggregates with tumour cells, shielding them from immune surveillance and high velocity shear forces, and facilitating extravasation to secondary sites while providing a rich source of growth factors^25,26,29^.

Investigating these interactions ex vivo is fundamental to understanding the association with hypercoagulation and tumour progression, considering firstly, treatment strategies and secondly, sub-phenotypes that clinically would fall under the same phenotype. We have previously shown in vitro that Tamoxifen pre-treatment of breast cancer cell lines augments their ability to induce platelet activation^30^ echoing clinical results^17,31^. However, our results have contrasted with laboratory studies where Tamoxifen either administered to patients or Tamoxifen pre-treatment of blood in vitro conversely inhibited platelet activation^32,33^. These contrasts are likely due to variance in methodological approaches and encourages further investigations. Clinically, Tamoxifen is administered orally or intravenously (IV), depending on the severity, staging and patient preference; allowing for availability within the circulation as well as ultimate accumulation of the therapy at the tumour site^5,34^. In order to mimic these scenarios, in this study we present two co-culture models making use of blood constituents (whole blood, platelet-rich plasma and platelet-poor plasma) and breast cancer cell lines (MCF-7 and T47D): in Model 1, breast cancer cell lines were treated with a physiological dose of Tamoxifen prior to exposure to untreated blood constituents; whereas in Model 2, blood was treated with Tamoxifen prior to exposure to untreated breast cancer cell lines. This allowed us to investigate hypercoagulation as defined by thrombin release and generation by breast cancer cells with ensuing effects on platelet activation identified by CD62P/P-selectin and lysosome membrane protein 3 (LAMP3)/CD63 expression, with corresponding platelet morphological alterations.

Additionally, we previously identified that breast cancer cell lines, MCF-7 and T47D, differentially induced platelet activation under Tamoxifen treatment^30^. Since these cell lines both correspond to the clinical luminal A subtype where ER and progesterone receptor (PR) expression is evident^35,36^, this was not entirely expected. Since ER status is predictive of better prognosis following treatment^37–39^, we investigated whether the ERs, ERα, most commonly studied^37,40^ and ERβ, considered as predictive marker of Tamoxifen resistance in breast tumours yet not clinically assessed^41–43^, showed any association with markers of hypercoagulation that could hold predictive value for thrombotic propensity relative to tumour phenotype.

## Results

### Non-tumorigenic breast epithelial cell line MCF10A induces low levels of coagulation and alters ER expression on exposure to blood

**Figure 1:**
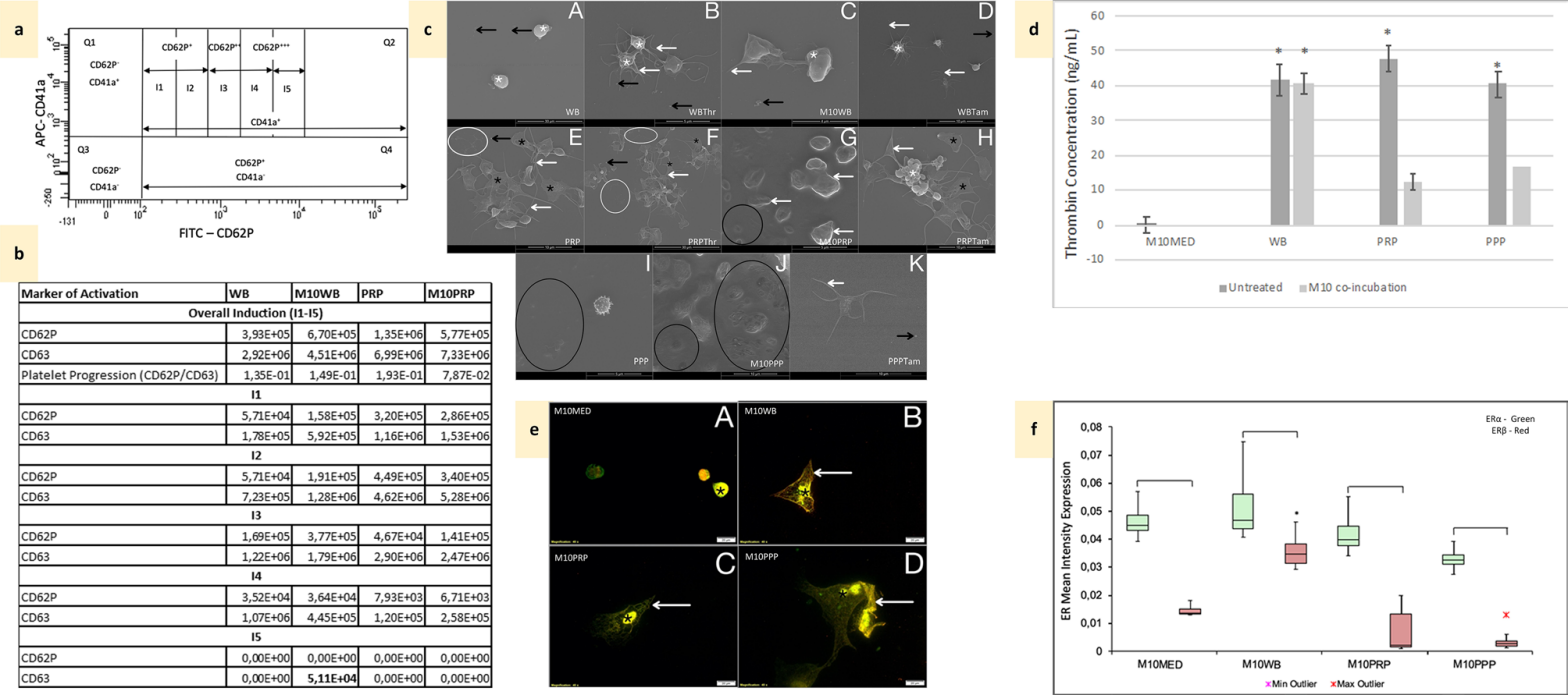
Controls; non-tumorigenic cell line MCF10A effects on coagulation and ER expression and Platelet ultrastructural alterations in Tamoxifen-treated blood constituents compared to controls. *WB* untreated whole blood, *M10WB* WB co-incubated with MCF10A cells, *PRP* untreated platelet-rich plasma, *M10PRP* PRP co-incubated with MCF10A cells, *PPP* Platelet-poor plasma, *M10PPP* PPP co-incubated with MCF10A cells, *M10MED* untreated MCF10A cells (media control). (**a**) Scatterplot diagram showing spread of platelet activation (CD62P or CD63) across defined interval gates. (**b**) Index of platelet activation (IPA) for CD62P and CD63, in blood constituents exposed to MCF10A cells. Overall and per interval gate levels. Bold: significantly (p < 0.05) different to matched control groups. (**c**) Platelet ultrastructural alterations. White*—membrane folds, black*—hyalomere spread, white arrow—extending filipodia, black arrow—microparticles, white circle—fibrin, black circle—fibrin clots with pores/plaques A: WB—inactive platelets with smooth membrane. B: WBThr Positive control WB (0.1 U/mL thrombin)—active platelets with membrane folds and extending filipodia. C: *M10WB* slightly active platelets. D: *WBTam* Tamoxifen-treated WB—active platelets with membrane folds, filipodia extensions and few microparticles E: PRP—spread platelets with pores, extending filipodia and fibrin F: *PRPThr* Positive control PRP (0.1 U/mL thrombin)—spread platelets aggregating, filipodia extension and fibrin formation. G: M10PRP—fibrin plaque formation with pores. H: *PRPTam* Tamoxifen-treated PRP—spread platelets aggregating, membrane budding, lamellipodia and filipodia extension. I: PPP—crenated red blood cells, platelet remnants and fibrin clots. J: M10PPP—fibrin plaque formation with pores. K: *PPPTam* Tamoxifen-treated PPP—scattered yet active platelets. (**d**) Thrombin concentrations *p < 0.05 compared to untreated MCF10A. (**e**) Co-localisation of ERα (green) and ERβ (red) in MCF10A cells. A: M10MED—nuclear ERα and ERβ, no cytoplasmic staining. B: M10WB—primarily nuclear ERα and cytoplasmic ERβ. C: M10PRP—nuclear (*) and cytoplasmic (white arrow) ERα expression. Low ERβ expression. D: M10PPP—high nuclear ERα and low ERβ in cytoplasm. (**f**) Box and whisker plots representing quantitative analysis of ERα (green) and ERβ (red) expression in MCF10A cells, following exposure to blood constituents; *significant (p < 0.05) differences compared to untreated MCF10A.

Co-incubation of blood constituents with MCF10A cells did not induce significant changes in IPA of either activation marker overall or per interval gate (Fig. 1a,b); except for (WB) CD63 IPA in I5 (Fig. 1b). Morphologically, platelets from untreated WB showed inactive platelets in resting phase (Fig. 1c-A); while platelets from thrombin-treated WB indicated activity, with the presence of membrane folds and extending filipodia (Fig. 1c-B). WB platelets exposed to MCF10A breast epithelial cells were mostly in resting phase, with some showing a smooth membrane with few surface folds and filipodia (Fig. 1c-C), however, this was lower than that of Tamoxifen-treated WB (Fig. 1c-D). This corresponds with detected thrombin levels which reflect that found in WB, since little to no levels of thrombin were detected in MCF10A-conditioned culture media (Fig. 1d). When PRP- (Fig. 1c-E) and PPP- (Fig. 1c-I), which are subjected to centrifugation steps concentrating fibrinogen, were exposed to MCF10A cells, fibrin plaque formation was evident (Fig. 1c-G,J), greater than that noted in the positive PRP control (Fig. 1c-F) and Tamoxifen-treated PRP (Fig. 1c-H) and PPP (Fig. 1c-K), explaining the reduction in detectable thrombin in these culture groups (Fig. 1d). In response to co-incubation with blood constituents, MCF10A cells changed from a typical epithelial morphology (Fig. 1e-A) to a star-shaped, elongated cell (Fig. 1e-B–D). Imaging cells showed higher ERα (green fluorescence) and lower ERβ (red fluorescence) concentrated within the nucleus on exposure to PRP and PPP (Fig. 1e-C,D), while exposure to WB increased cytoplasmic ERβ (Fig. 1e-B) (DAPI nuclear stain not shown since it obscures nuclear ER expression). This was verified quantitatively, with some variable expression in response to blood constituents (Fig. 1f).

### PRP presents with higher thrombin concentration and platelets in later stages of activation than WB, but remain responsive to induced coagulation

**Figure 2:**
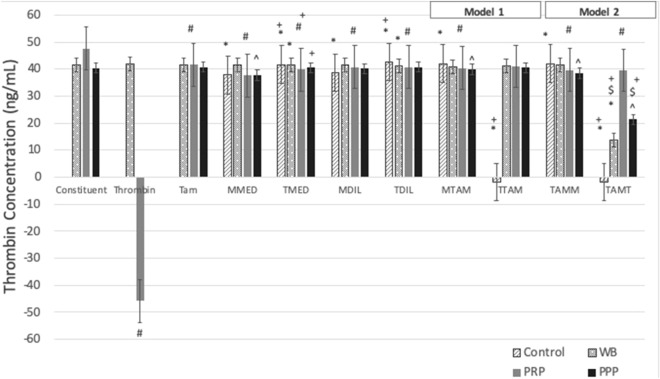
Bar graph showing thrombin generation in cumulative (Model 1) or circulatory (Model 2) Tamoxifen-therapy. Constituent: WB—untreated whole blood, PRP—untreated platelet-rich plasma, PPP—untreated platelet-poor plasma. Thrombin: Positive controls—WB and PRP incubated with 0.1 U/mL of thrombin. Tam: Tamoxifen-treatment of blood constituents (WB/PRP/PPP). Cells: M—MCF7 cells, T—T47D cells. M/TMED: conditioned media control of MCF7/T47D cells. M/TDIL: diluent control of MCF7/T47D cells. Model 1: M/T TAM: Tamoxifen-treated MCF7/T47D cells. Model 2: TAM M/T: Tamoxifen-treated blood constituents exposed to MCF7/T47D cells. *p < 0.05 compared to untreated WB; ^#^p < 0.05 compared to untreated PRP; ^^^p < 0.05 compared to untreated PPP. ^$^p < 0.05 between matched cell lines compared between models.

Baseline levels of platelet activation were determined by thrombin activity (Fig. 2), IPA (Table 1) and platelet ultrastructure. Thrombin activity assessment of blood constituent control samples indicated baseline levels of thrombin availability in WB at 41.7 ng/mL ± 3.58 (Fig. 2). PPP had a slightly decreased level of thrombin availability, at 40.4 ng/mL ± 2.37; and was assayed for the presence of platelet microparticles; expressing CD62P IPA at 1.44E+05 ± 2.04E+05 and CD63 IPA 5.35E+05 ± 3.00E+05. PRP indicated significantly (p < 0.05; p = 1.73E-06) greater levels of thrombin availability 47.7 ng/mL ± 1.01E+03. This reflects the significantly higher early stage (CD62P, released initially from α granules) and late stage (CD63, released later from δ granules) compared to WB as expected, given centrifugation steps (Table 1). A ratio between CD62P IPA and CD63 IPA was used to explain the capacity of platelets to progress to later stages of activation; where a lower ratio indicated a later stage as well as a greater spread throughout interval gates (Supplementary Tables S1, S2), as identified in PRP. This was also evident in micrographs (Fig. 1c-E). This concept is further noted in the positive controls, where the addition of suprathreshold thrombin to WB induced only slightly higher overall CD62P but significantly higher CD63 IPA (Table 1). Thrombin availability increased only slightly (p > 0.05) and this could be attributed to the binding of the exogenous thrombin to the platelets within the WB itself (Fig. 2). The addition of thrombin to PRP as a positive control; however, significantly heightened levels of thrombin availability (p = 1.17E−06), much higher than the detectable levels of the assay itself, resulting in a negative value of − 45.8 ng/mL ± 2.29E+02^44,45^. Since PRP contains high numbers of platelets this allows for interaction rates with thrombin to be heightened as platelet activation proceeds (Fig. 2). Platelets at this heightened level of expression lose CD62P, however, CD63 persists, reflecting hypercoagulation (Table 1). Morphologically this is seen as platelet aggregation, with spread platelets, filipodia extension, and more microparticles and fibrin formation (Fig. 1c-F).

**Table 1:** Index of platelet activation (IPA) for markers CD62P and CD63, and platelet progression (CD62P/CD63 ratio) in blood constituents; WB/PRP exposed to Tamoxifen-treated breast cancer cells (Model 1) and Tamoxifen-treated blood constituents; WB/PRP exposed to breast cancer cells (Model 2).

Marker of activation	Controls							Model 1		Model 2	
	WB	WBThr	WBTam	MMEDWB	TMEDWB	MDILWB	TDILWB	MTAMWB	TTAMWB	WBTAMM	WBTAMT
**Q2 (I1–I5)**											
CD62P	3.93E+05 ± 1.72E+05	4.99E+05 ± 1.13E+05	4.93E+05 ± 2.07E+05	**6.33E+05** ± 2.52E+05	4.31E+05 ± 1.73E+05	5.48E+05 ± 2.43E+05	3.63E+05 ± 1.99E+05	**6.24E+05** ± 1.67E+05	7,78E+05* ± 3,96E+05	**6,26E+05** ± 1,56E+05	6,01E+05* ± 2,37E+05
CD63	2.92E+06 ± 8.06E+05	**3.35E+06** ± 9.00E+05	3.01E+06 ± 1.58E+06	3.65E+06 ± 1.56E+06	**4.08E+06** ± 1.35E+06	3.15E+06 ± 1.28E+06	2.80E+06 ± 1.97E+06	3.07E+06 ± 1.38E+06	**4,87E+06** ± 2,36E+06	3,64E+06 ± 9,63E+05	**3,69E+06** ± 1,68E+06
Platelet Progression (CD62P/CD63)	1.35E−01 ± 0.016	1.49E−01 ± 0.013	1.64E−01 ± 0.026	1.74E−01 ± 0.019	1.06E−01 ± 0.019	1.74E−01 ± 0.020	1.30E−01 ± 0.015	2.03E−01 ± 0.026	1,60E−01 ± 0,022	1,72E−01 ± 0,019	1,63E−01 ± 0,036

Marker of activation	Controls							Model 1		Model 2	
	PRP	PRPThr	PRPTam	MMEDPRP	TMEDPRP	MDILPRP	TDILPRP	MTAMPRP	TTAMPRP	PRPTAMM	PRPTAMT
**Q2 (I1–I5)**											
CD62P	**1.35E+06**^**#**^ ± 1.79E+05	7.60E+05^#^ ± 1.44E+05	7.73E+05 ± 1.67E+05	1.20E+06 ± 5.24E+05	1.20E+06 ± 5.05E+05	7.07E+05 ± 4.57E+05	*1.76E+06*^*#*^ ± 4.72E+05	8.62E+05^#^ ± 6.04E+05	*2,08E+06*^*#*^ ± 6,27E+05	7,16E+05^#^ ± 2,73E+05	*1,52E+06* ± 2,52E+05
CD63	**6.99E+06**^**#**^ ± 2.53E+06	8.41E+06^#^ ± 1.35E+06	9.04E+06^#^ ± 1.55E+06	1.25E+07^#^ ± 3.80E+06	*8.39E+06*^*#*^ ± 4.07E+06	7.93E+06 ± 3.57E+06	*1.14E+07*^*#*^ ± 4.15E+06	6.11E+06^#^ ± 4.66E+06	*1,23E+07*^*#*^ ± 4,87E+06	7,92E+06^#^ ± 2,28E+06	*1,01E+07*^*#*^ ± 1,95E+06
Platelet Progression (CD62P/CD63)	1.53E−01 ± 0.020	9.04E−02^#^ ± 0.022	8.55E−02^#^ ± 0.015	*9.62E−02*^*#*^ ± 0.015	1.43E−01 ± 0.022	8.92E−02^#^ ± 0.023	*1.54E−01* ± 0.011	1.41E−01^#^ ± 0.028	1,69E−01 ± 0,024	9,03E−02 ± 0,025	1,50E−01 ± 0,024

Overall levels (Q2; I1–I5) were assessed.

Bold p < 0.05 compared to untreated WB; *p < 0.05 compared to matched cell line exposed to WB (M/T MEDWB); italics p < 0.05 compared to matched cell lines between Models; underline p < 0.05 compared to untreated PRP; ^#^p < 0.05 compared to matched cell line exposed to PRP (M/T MEDPRP); ± standard error of mean.

*WB* untreated whole blood, *WBThr* positive control incubated with 0.1 U/mL thrombin, *WBTam* Tamoxifen-treated WB, *Cells* M—MCF7, T—T47D, *Model 1: M/T MEDWB* WB exposed to MCF7/T47D cells, *M/T DILWB* WB exposed to diluent-treated MCF7/T47D cells, *M/T TAMWB* WB exposed to Tamoxifen-treated MCF7/T47D cells, *Model 2: WBTAM M/T* Tamoxifen-treated WB exposed to MCF7/T47D cells.

### Breast cancer cell lines induce a hypercoagulatory environment

**Figure 3:**
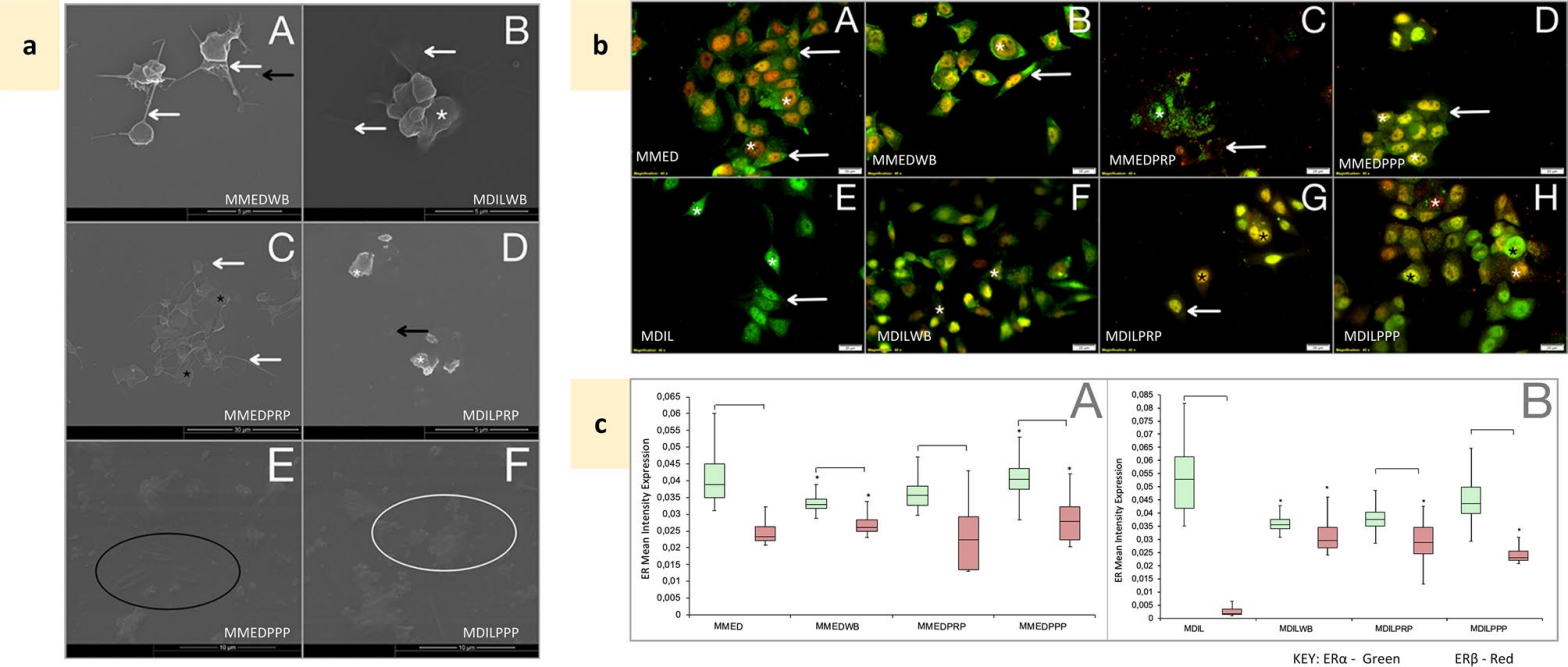
Effects of blood constituent incubation with MCF7 cells—controls. *MMED* media-treated MCF7 cells, *MMEDWB* WB co-incubated with media-treated MCF-7 cells, *MMEDPRP* PRP co-incubated with media-treated MCF-7 cells, *MMEDPPP* PPP co-incubated with media-treated MCF-7 cells, *MDIL* diluent-treated MCF7 cells, *MDILWB* WB co-incubated with diluent-treated MCF-7 cells, *MDILPRP* PRP co-incubated with diluent-treated MCF-7 cells, *MDILPPP* PPP co-incubated with diluent-treated MCF-7 cells. (**a**) Platelet ultrastructural alterations. White*—membrane folds, black*—hyalomere spread, white arrow—extending filipodia, black arrow—microparticles, white circle—fibrin, black circle—fibrin pores A: MMEDWB—active platelets, smooth membrane, extending filipodia and microparticles. B: MDILWB—platelets with membrane folds and filipodia. C: MMEDPRP—spread platelets and filopodia. D: MDILPRP—platelets with membrane folds and microparticles. E: MMEDPPP and F: MDILPPP—remnants of platelets, fibrin and pores. (**b**) Co-localisation of ERα (green) and ERβ (red) in MCF7 cells, following exposure to blood constituents. A: MMED—primarily cytoplasmic (white arrow) ERα and nuclear (*) ERβ expression. B: MMEDWB—cellular processes extend, cytoplasmic ERα and nuclear ERβ. C: MMEDPRP—diffuse ERα and ERβ expression. D: MMEDPPP—high cytoplasmic ERα expression and ERβ contained within the nucleus. E: MDIL—nuclear and cytoplasmic ERα, some nuclear ERβ. F: MDILWB—nuclear and cytoplasmic ERα, greater nuclear ERβ expression. G: MDILPRP and H: MDILPPP—high ERα and ERβ nuclear expression, minimal cytoplasmic expression. (**c**) Box and whisker plots representing quantitative analysis of ERα (green) and ERβ (red) expression in MCF7 cells, following exposure to blood constituents. A: Conditioned media-treated MCF7 cells. B: Diluent-treated MCF7 cells (0.1% DMSO). ^[^p < 0.05 between ERα and ERβ within the treatment group. *p < 0.05 between matched ERs compared to untreated MCF7.

**Figure 4:**
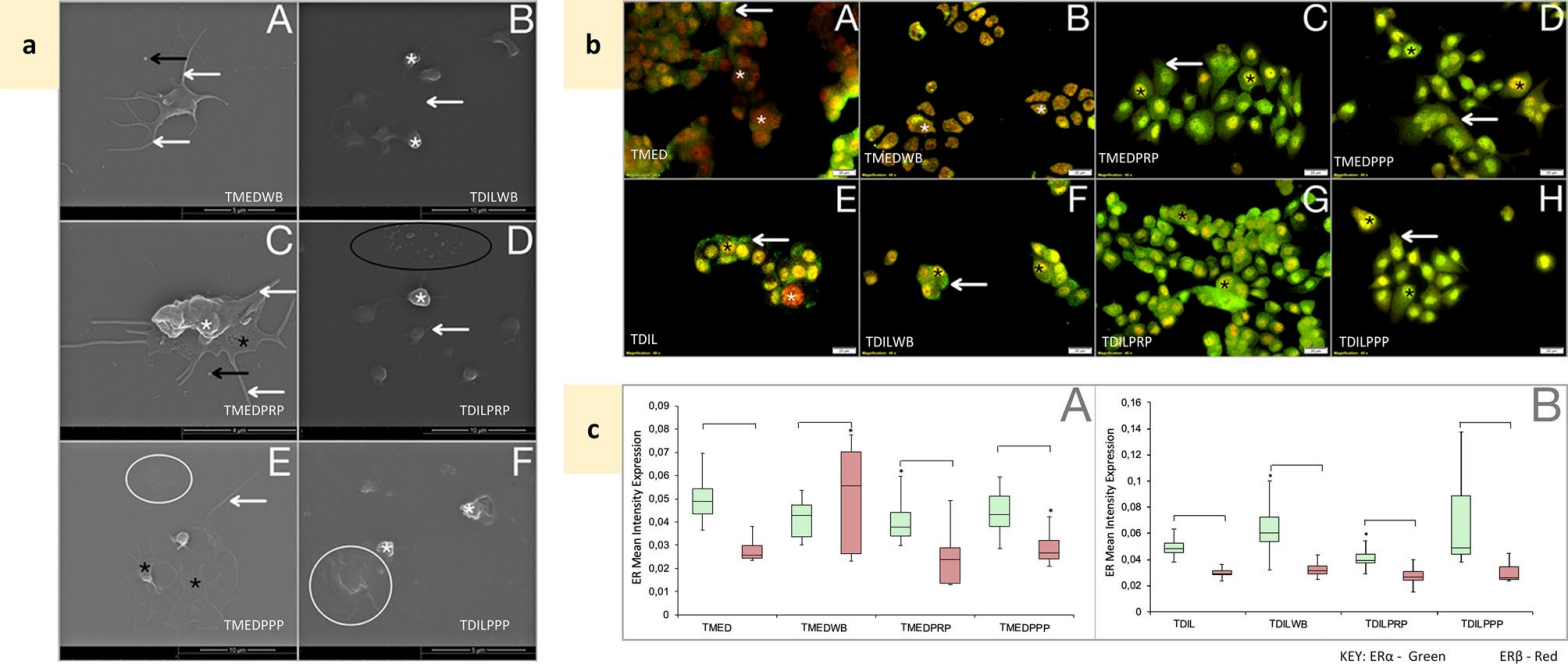
Effects of blood constituent incubation with T47D cells—controls. *TMED* media-treated T47D cells, *TMEDWB* WB co-incubated with media-treated T47D cells, *TMEDPRP* PRP co-incubated with media-treated T47D cells, *TMEDPPP* PPP co-incubated with media-treated T47D cells, *TDIL* diluent-treated T47D cells, *TDILWB* WB co-incubated with diluent-treated T47D cells, TDILPRP—PRP co-incubated with diluent-treated T47D cells, *TDILPPP* PPP co-incubated with diluent-treated T47D. (**a**) Platelet ultrastructural alterations. White *—membrane folds, black *—platelet hyalomere spread, white arrow—extending filipodia, black arrow—microparticles, white circle—fibrin, black circle—fibrin deposits. A: TMEDWB—slightly active platelet with filipodia and microparticles; B: TDILWB—mostly resting platelets; C: TMEDPRP—spread platelet, filipodia and microparticles. D: TDILPRP—platelets with smooth membrane and extending filipodia, with microparticles. E: TMEDPPP: fully spread platelets with fibrin deposits. F: TDILPPP: fibrin plaque formation. (**b**) Co-localisation of ERα (green) and ERβ (red) in T47D cells, following exposure to blood constituents. A: TMED: primarily cytoplasmic ERα (white arrow) and ERβ expression in the nucleus (*). B: TMEDWB: ERα and ERβ expression in the nucleus (*). C: TMEDPRP: cytoplasmic and nuclear ERα expression (arrow) and nuclear ERβ (*). D: TMEDPPP: high cytoplasmic ERα (white arrow) and nuclear ERβ (*). E: TDIL—diffuse cytoplasmic and nuclear ERα (white arrow), nuclear ERβ (*). F: TDILWB—cytoplasmic and nuclear ERα (white arrow), higher nuclear ERβ (*). G: TDILPRP—cytoplasmic and nuclear ERα and nuclear ERβ (*). H: TDILPPP—high nuclear and cytoplasmic ERα (black *) and higher nuclear ERβ (white *). (**c**) Box and whisker plots representing quantitative analysis of ERα (green) and ERβ (red) expression in T47D cells, following exposure to blood constituents. A: Conditioned media-treated T47D cells. B: Diluent-treated T47D cells (0.1% DMSO). ^[^Significant differences between ERα and ERβ within the treatment group. *Significant (p < 0.05) differences between matched ERs compared to untreated T47D.

Assessment of thrombin generation showed that control, untreated breast cancer cells secreted more thrombin; MCF7 37.77 ng/mL ± 4.00E+01; T47D 41.61 ng/mL ± 1.01E+03, than the non-tumorigenic MCF10A cells; 0.073 ng/mL ± 6.59E+01 (Figs. 1d, 2). MCF7 cells generated significantly less thrombin than the WB control (p = 7.08E−17); however, MCF7 cells exposed to WB increased the level of detectable thrombin significantly (p = 1.17E−06) when compared to untreated MCF7 cells. This greater level of available thrombin could be due to low levels of platelet activation evident in untreated WB (Fig. 3a-A). However, when PRP and PPP were exposed to MCF7 cells, slight decreases in detectable thrombin levels is noted compared to matched blood constituents (Fig. 2), possibly due to platelet activation within the concentrated PRP as well as the generation of fibrin in both samples (Fig. 3a-C,E). T47D cells secreted thrombin at 41.61 ng/mL ± 1.01E+03 which lowered significantly (p = 1.30E−06) when cells were exposed to blood constituents (Fig. 2), attributable to platelet ligation and subsequent activation. Both breast cancer cell lines induced higher CD62P and CD63 IPA in WB than the non-tumorigenic MCF10A cell line (p > 0.05); with MCF7 cells inducing significantly higher CD62P, and T47D cells induced significantly more CD63 IPA, compared to WB (Table 1). Matched diluent control-induction of IPA (Table 1) and thrombin generation was not different to untreated cell lines (Fig. 2), and platelets remained in early stages of activation (Figs. 3a-B,D,F, 4a-B,D,F). In PRP, a consistently higher level of platelet activation was noted. PRP exposed to MCF7 cells (Table 2) showed an increase in CD62P IPA and CD63 IPA, shifting to later stages of activation (p < 0.05) which was also visually evident (Figs. 3a-C, 4a-C). PRP exposed to T47D cells (Table 1) showed a decrease in CD62P IPA and an increase in CD63 IPA, with a slight shift to later stage activation, apparent in ultrastructural assessment (Fig. 4a-C), with WB exposure inducing lower levels of activation (Fig. 4a-A). While PPP exposure to T47D cells indicated the presence of fibrin, with some platelets still evident (Fig. 4a-E).

### Effects of cumulative Tamoxifen (Model 1) on hypercoagulation

**Figure 5:**
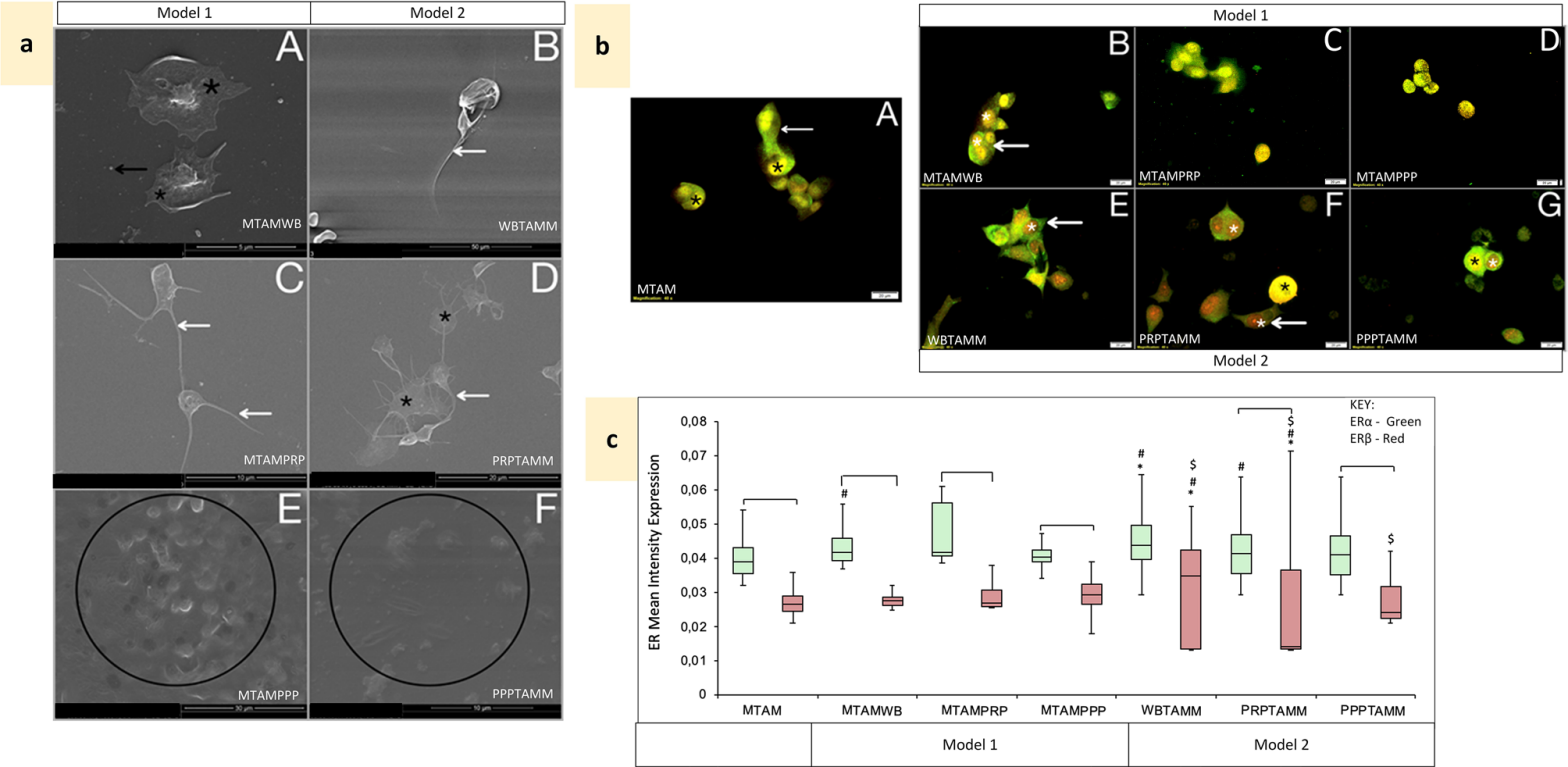
Effects of Tamoxifen treatment, cumulative (Model 1) and circulatory (Model 2) following co-incubation with MCF7 cells. *MTAM* Tamoxifen-treated MCF7 cells. *MTAMWB* Tamoxifen-treated MCF7 cells co-incubated with WB. *MTAMPRP* Tamoxifen-treated MCF7 cells co-incubated with PRP. *MTAMPPP* Tamoxifen-treated MCF7 cells co-incubated with PPP. *WBTAMM* Tamoxifen-treated WB co-incubated with MCF7 cells. *PRPTAMM* Tamoxifen-treated PRP co-incubated with MCF7 cells. *PPPTAMM* Tamoxifen-treated PPP co-incubated with MCF7 cells. (**a**) Platelet ultrastructural alterations. Black *—platelet hyalomere spread, white arrow—extending filipodia, black arrow—microparticles, black circle—fibrin clots and plaques with pores. A: MTAMWB—active platelets with a hyalomere spread and microparticles. B: WBMTAM—active platelets with a folded membrane and extending filipodia. C: MTAMPRP—platelets with a hyalomere spread and extending filipodia. D: PRPTAMM—platelets in early stages of aggregation, with extending filipodia. E: MTAMPPP—fibrin plaque formation with some pores. F: PPPTAMM—fibrin pores and remnants of fibrin. (**b**) Co-localisation of ERα (green) and ERβ (red) in MCF7 cells; following Tamoxifen-treatment and exposure to blood constituents (Model 1) and Tamoxifen-treatment of blood constituents exposed to MCF7 cells (Model 2). A: MTAM—cytoplasmic and nuclear ERα (arrow) and nuclear ERβ (*). B: MTAMWB—nuclear and cytoplasmic ERα (arrow) and nuclear ERβ (*). C: MTAMPRP—nuclear and low cytoplasmic ERα (arrow) and nuclear ERβ (*). D: MTAMPPP—low cytoplasmic and high nuclear ERα (arrow) and nuclear ERβ (*). E: WBTAMM—high cytoplasmic ERα (arrow) and extremely high nuclear ERβ (*). F: PRPTAMM—low cytoplasmic ERα (arrow) and high nuclear ERβ (white *), combined expression is noted (black *). G: PPPTAMM—cytoplasmic ERα with nuclear of ERβ (*). **(c)** Box and whisker plots representing quantitative analysis of ERα (green) and ERβ (red) expression in MCF7 cells for Model 1 and 2. ^[^p < 0.05 between ERα and ERβ within the treatment group. ^#^p < 0.05 between matched ERs compared to untreated MCF7. *p < 0.05 between matched ERs compared to Tamoxifen-treated MCF7. ^$^p < 0.05 between matched ERs and blood constituent groups compared between models.

Cumulative effects of Tamoxifen were mimicked by pre-treating cancer cell lines (Model 1) prior to co-incubation with blood constituents. Tamoxifen-treated MCF7 breast cancer cells (Model 1) induced significantly greater secretion of thrombin (p = 1.7E−06) when compared to conditioned media from the untreated MCF7 cell line control (Fig. 2). This was reduced on exposure to all blood constituents, indicative of platelet activation, in WB (Fig. 5a-A) and PRP (Fig. 5a-C) samples, and fibrin formation in PPP (Fig. 5a-E). In WB exposed Tamoxifen-treated MCF7 cells, CD63 and CD62P IPA increased, the latter significantly compared to untreated WB and comparable to that of WB exposed to untreated MCF7 cells (Table 1). Moreover, platelet activation was in a later stage (p > 0.05) (Table 1), with corresponding morphology showing spreading of the hyalomere (Fig. 5a-A). This contrasted with PRP exposed to Tamoxifen-treated MCF7 cells where CD63 and CD62P IPA was lower compared to untreated PRP (Table 1) reflecting platelets at an earlier stage of activation with pseudopodia extension and the presence of microparticles and fibrin (Fig. 5a-C).

**Figure 6:**
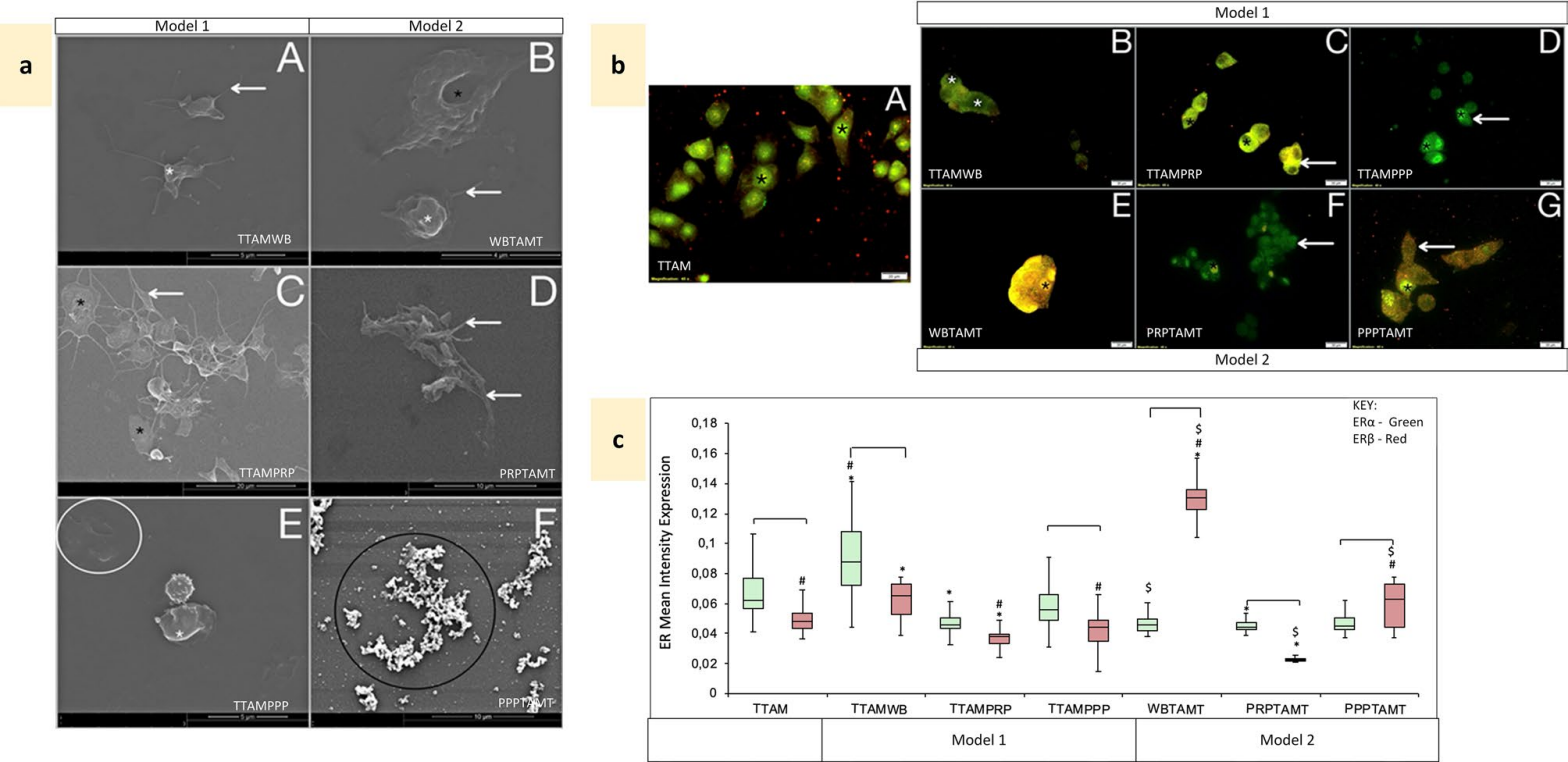
Effects of Tamoxifen treatment, cumulative (Model 1) and circulatory (Model 2) following co-incubation with T47D cells. *TTAM* Tamoxifen-treated T47D cells. *TTAMWB* Tamoxifen-treated T47D cells co-incubated with WB. *TTAMPRP* Tamoxifen-treated T47D cells co-incubated with PRP. *TTAMPPP* Tamoxifen-treated T47D cells co-incubated with PPP. *WBTAMT* Tamoxifen-treated WB co-incubated with T47D cells. *PRPTAMT* Tamoxifen-treated PRP co-incubated with T47D cells. *PPPTAMT* Tamoxifen-treated PPP co-incubated with T47D cells. (**a**) Platelet ultrastructural alterations. White *—membrane folds, black *—platelet hyalomere spread, white arrow—extending filipodia, black arrow—microparticles, white circle—fibrin, black circle—fibrin deposits. A: TTAMWB—active platelets with a folded membrane and extending filipodia. B: WBTAMT—active platelets with a folded membrane and extending filipodia with fibrin deposits and pores C: TTAMPRP—platelets with a hyalomere spread and extending filipodia. D: PRPTAMT—platelets with extending filipodia. E: TTAMPPP—fibrin deposits and plaque formation with some pores. F: PPPTAMT—fibrinogen deposits. (**b**) Co-localisation of ERα (green) and ERβ (red) in T47D cells; following Tamoxifen-treatment and exposure to blood constituents (Model 1) and Tamoxifen-treatment of blood constituents exposed to MCF7 cells (Model 2). A: TTAM—cytoplasmic and nuclear ERα (*) and ERβ dispersed. B: TTAMWB—cytoplasmic ERα and nuclear ERβ with some nuclei devoid of expression (*). C: TTAMPRP—low cytoplasmic ERα (arrow) and cytoplasmic ERβ, with the nucleus devoid of expression (*). D: TTAMPPP—low cytoplasmic and high nuclear ERα (arrow) with nuclear ERβ (*). E: WBTAMT—cytoplasmic ERα and extremely high nuclear ERβ (*). F: PRPTAMT—low cytoplasmic ERα (arrow) with nuclear ERα and ERβ (*). G: PPPTAMT—cytoplasmic and nuclear ERα (*), and cytoplasmic ERβ (arrow). (**c**) Box and whisker plots representing quantitative analysis of ERα (green) and ERβ (red) expression in T47D cells for Model 1 and 2. ^[^p < 0.05 between ERα and ERβ within the treatment group. ^#^p < 0.05 between matched ERs compared to untreated T47D. ^*^p < 0.05 between matched ERs compared to Tamoxifen-treated T47D. ^$^p < 0.05 between matched ERs and blood constituent groups compared between models.

Tamoxifen-treated T47D cells significantly heightened levels of thrombin generation (p = 1.30E−06) much higher than the detectable levels of the assay itself, resulting in a negative value of − 1.83 ng/mL ± 2.78E+00, as seen in the positive control PRP exposed to thrombin (Fig. 2). When compared to matched untreated T47D cells exposed to blood constituents; Tamoxifen pre-treatment of cells exposed to WB reduced thrombin detection (Fig. 2); however, early platelet activation was evident with a significant increase in CD62P IPA (p = 0.046) and CD63 IPA (p = 0.04) and corresponding extension of pseudopodia, spread of the hyalomere and microparticle release (Table 1, Fig. 6a-A). PRP exposure to Tamoxifen-treated T47D cells significantly increased thrombin generation (Fig. 2) and CD62P IPA and CD63 IPA (Table 1). Platelet activation indicated a shift to a later stage (Table 1); with platelets presenting spread hyalomeres, extending lamellipodia and filipodia, and early aggregation (Fig. 6a-C). Both PRP and PPP induced fibrin formation (Fig. 6a-C,E). The induction of hypercoagulation, was thus more evident by the T47D cell line.

### Effects of circulating Tamoxifen (Model 2) on hypercoagulation

The effects of pre-treatment of whole blood with Tamoxifen (and subsequent separation into blood constituents) on coagulation was first assessed. Thrombin availability was significantly reduced only in PRP samples (p = 1.17E−06) (Fig. 2) and increased overall CD62P and CD63 IPA in WB (p > 0.05) and PRP (p < 0.05; p = 0.03), detected (Table 1). This indicated that platelet activation remained within early stages in Tamoxifen-treated WB (Table 1) while derived PRP showed a shift to a later stage of platelet activation (Table 1) with platelets more active and aggregated than those from WB (Figs. 5a, 6a).

Tamoxifen pre-treated blood constituents were then exposed to untreated breast cancer cells to mimic circulating Tamoxifen (Model 2). When compared to Model 1, pre-treated WB exposed to untreated MCF7 cells induced a slightly higher level of thrombin generation (Fig. 2), with comparable levels of CD62P and CD63 IPA indicating early activation (Table 1, Fig. 5a-B). In Tamoxifen-treated PRP and PPP exposed to untreated MCF7 cells significantly lower thrombin was detected (p = 1.30E−06) (Fig. 2), reflecting binding and subsequent late stage platelet activation and aggregation in PRP (Table 1, Fig. 5a-D) and fibrin formation in PPP (Fig. 5a-F).

Thrombin activity in Tamoxifen-treated WB exposed to T47D cells was low; however, this is potentially due the binding of thrombin to the cells themselves (Fig. 6). Platelet activation was confirmed with both CD62P and CD63 IPA significantly heightened compared to untreated WB, similar to that of Model 1 (Table 1), with active platelets extending filipodia and fibrin deposits evident (Fig. 6a-B). Thrombin generation in Tamoxifen-treated PRP and PPP samples was also reduced compared to matched samples (Fig. 2). In PRP, this reflected corresponding low CD62P and CD63 IPA (Table 1), and platelet aggregation with platelets appearing exhausted (Fig. 6a-D). Notably in PPP, fibrinogen deposits reflected an inability of cells to mediate fibrin formation even though thrombin was present (Figs. 2, 6a-F).

### ER expression in breast cancer cell lines alter under mediation by blood constituents

MCF7 cells typically have an epithelial-like morphology with thicker cellular processes (Fig. 3b-A). In untreated MCF7 cells, ERα (green fluorescence) is primarily cytoplasmic, with ERβ (red fluorescence) nuclear (Fig. 3b-A). Quantitative assessment showed overall, greater ERα expression than ERβ (Fig. 3c-A), even under exposure to blood constituents. On exposure to WB, ERα expression decreased (p = 3.16E−50) while ERβ increased (p = 5.5E−20) (Fig. 3b-B,c-A), significantly. A similar trend was observed under exposure to PRP; however, dispersed fluorescence was noted (Fig. 3b-C); but when exposed to PPP, both ERs increased significantly (p = 4.08E−16) (Fig. 3c-A), with heightened ERα intensity in the nucleus (Fig. 3b-D). The diluent control showed similar expression profiles, with lower ERβ (Fig. 3b-E–H,c-B).

T47D cells demonstrate a highly cohesive cobblestone appearance, with ERα similarly cytoplasmic and ERβ mostly nuclear; however, ERβ expression is consistently higher compared to that of MCF7 cells (p > 0.05) (Fig. 4b-A,c-A). Exposure to WB resulted in translocation of ERα to the nucleus (Fig. 4b-B), with variable and significantly higher ERβ expression (p = 7.04E−06); similarly, PPP induced an increase in ERβ albeit not as high (Fig. 4b-D). Levels of both markers were reduced on exposure to PRP (Fig. 4b-C). Notably, the diluent control showed some variation and as such is presented (Fig. 4b-E–H,c-B).

### Tamoxifen alters ER expression in both models

In Model 1, in which blood constituents were co-cultured with Tamoxifen-treated MCF7 cells, ERα expression (primarily cytoplasmic) was consistently higher than ERβ (primarily nuclear) (Fig. 5b-B–D,c) compared to Tamoxifen-treated MCF7 cells (Fig. 5b-A). However, Tamoxifen-treated whole blood exposed to untreated MCF7 cells (Model 2) (Fig. 5b-E), significantly heightened ERα and ERβ expression (Fig. 5c), in the perinuclear and nuclear regions, respectively. This trend was similar to that induced by Tamoxifen-treated PPP (Fig. 5b-G). Conversely, Tamoxifen-treated PRP induced significantly reduced ERβ (p = 1.16E−21), although considerable variation within the nucleus was noted (Fig. 5b-F,c).

Tamoxifen treatment induced higher ERα expression in T47D cells (Fig. 6b-A,c), similar to its effects on MCF7 cells (Fig. 5b-A,c). WB exposure to Tamoxifen pre-treated T47D cells (Model 1) increased ERα (p = 1.48E−25) and ERβ (p = 2.85E−42) expression significantly (Fig. 6c), in the cytoplasm and nucleus respectively (Fig. 6b-B). PRP exposure caused translocation of ERα to the nucleus and ERβ to the cytoplasm (Fig. 6b-C); however, mean intensity dropped significantly (Fig. 6c). PPP exposure followed a similar pattern (Fig. 6b-D,c). In Model 2, T47D cells exposed to Tamoxifen-treated WB significantly heightened nuclear ERβ (p = 1.38E−06) expression and reduced ERα (although some cells show high intensity spots) (Fig. 6b-E), Tamoxifen-treated PPP followed a similar trend (Fig. 6b-G,c). This contrasted with the effects of Tamoxifen-treated PRP where both ERs were reduced (Fig. 6b-F).

When assessing matched groups (Figs. 5c, 6c), overall the MCF7 cell line differentially responded to blood constituents, whereas the T47D cell line at least in Model 1, responded by primarily increasing ERβ expression.

### Weak correlation between hypercoagulation and ER expression

Overall, significant (p < 0.05), positive (r > 0.5) correlation was found between CD62 and CD63 expression; while significant correlations were also found between ERβ, ERα and markers of platelet activation, they remained weak (Table 2).

**Table 2:** Tabulation showing r values obtained from the nonparametric Spearman’s correlation between CD62P, CD63 IPA and ERα and ERβ, with thrombin detection for MCF7and T47D cells.

Spearman rank order correlations Highlighted correlations are significant at p < 0.05. r = 0.6																	
	Overall	Control				Model 1						Model 2					
		MMEDWB	MMEDPRP	TMEDWB	TMEDPRP	MCF7 Overall	T47D Overall	MTAMWB	MTAMPRP	TTAMWB	TTAMPRP	MCF7 Overall	T47D Overall	WBTAMM	PRPTAMM	WBTAMT	PRPTAMT
**Thrombin generation**																	
CD62P IPA	− 0.075	− 0.314	0.0857	− 0.028	0.257	− 0.242*	0.1319	− 0.2	0.7714	0.3	0.3142	− 0.24795*	− 0.06761	− 0.6572	− 0.486	− 0.579	0.4
CD63 IPA	*− 0.047*	− 0.314	*0.0285*	*0.314*	*0.428*	*− 0.195*	*0.1018*	*− 0.429*	0.771	− 0.7	− 0.086	*− 0.17414*	*− 0.03075*	0.8986*	− 0.4857	0.6	0.1
ER-Alpha	0.016	− 0.714	0.0294	0.257	0.25	*0.16584*	*0.0425*	0.4857	− 0.4857	0.3	0.1426	*0.173056*	0.01357	0.3142	***0.7714***	− 0.657	− 0.1
ER-Beta	− 0.189*	− 0.2	− 0.264	− 0.257	**0.142**	*− 0.00069*	*− 0.037*	0.485	− 0.1428	0.4	− 0.314	*− 0.2009*	− 0.10588	0.4286	***− 0.6***	− 0.486	− 0.6

*MMEDWB* MCF7 cells exposed to WB, *MMEDPRP* MCF7 cells exposed to PRP, *MTAMWB* Tamoxifen-treated MCF7 cells exposed to WB, *MTAMPRP* Tamoxifen-treated MCF7 cells exposed to PRP, *WBTAMM* MCF7 cells exposed to Tamoxifen-treated WB, *PRPTAMM* MCF7 cells exposed to Tamoxifen-treated PRP, *TMEDWB* T47D cells exposed to WB, *TMEDPRP* T47D cells exposed to PRP, *TTAMWB* Tamoxifen-treated T47D cells exposed to WB, *TTAMPRP* Tamoxifen-treated T47D cells exposed to PRP, *WBTAMT* T47D cells exposed to Tamoxifen-treated WB, *PRPTAMT* T47D cells exposed to Tamoxifen-treated PRP.

*p < 0.05 compared to thrombin detection, italics p < 0.05 compared to CD62P IPA, bold p < 0.05 compared to CD63 IPA.

## Discussion

Modelling the clinical environment ex vivo allows investigation of cellular responses with reduced interference but is also limited by varying methodologies and approaches that may affect outcome. In the tumour microenvironment, platelets play a key role in priming tumour cells for metastasis, even before clinical diagnosis^46^, and in the metastatic process itself^25,26,28,29^. During this process, changes in coagulatory mechanisms may lead to thromboembolic complications^3^. Tamoxifen is known clinically, to increase risk of a thromboembolic event^2,47^, but laboratory investigations provide contradictory results, most likely due to varying methodologies^30,32,33^. Furthermore Tamoxifen is also used as a chemopreventive agent^48,49^, and despite published clinical studies indicating its association with hypercoagulation^7–9^, the risk of thrombosis is not readily considered in future clinical scenarios. We mimicked accumulation of Tamoxifen at the tumour site (Model 1), and recreated intravenously administered Tamoxifen (Model 2)^5^, in addition to testing blood constituents. In both models we identified that platelets in PRP were more active, shown morphologically and by expression of CD62P and CD63 markers, than those derived from whole blood. However, these platelets remained responsive due to PRP being generated by soft centrifugation^50^. PRP did elicit more thrombin via granular release^51^, than WB, which compounded breast cancer-cell induced hypercoagulation.

A hypercoagulable state, also known as a prothrombotic state, in malignant cancers occurs when tumour cells activate the coagulation system and cause thrombi, formed by intravascular platelet aggregates^52,53^. We show that breast cancer cell lines induce a hypercoagulatory environment mediated by secretion of thrombin, a procoagulant factor. Thrombin is not readily generated by the non-tumorigenic cell line, MCF10A, as shown by our results echoing other studies which also indicate that MCF10A express low to no PAR1 receptors^54–56^. This suggests that the low levels of platelet activation and fibrin formation we identified were dependent on a different agonist, potentially Tissue Factor^57^. We also found that on exposure to blood constituents, MCF10A cells alter ERα and ERβ expression. Interestingly, studies suggest MCF10As present with a basal-like phenotype^35^, capable of expressing luminal markers^55^ or are undergoing epithelial-mesenchymal transition^58^; thereby raising the question of whether these cells are an effective model of normal breast epithelial cells.

Expression of ER defines luminal phenotype cancers, which typically would be treated with hormone-therapy; pre-menopausal patients would undergo first-line treatment with Tamoxifen, regarded as the ‘gold standard’^59,60^. Studies suggest that hormone-therapies induce heightened sensitivity to thrombin by upregulating PAR receptors, further increasing thromboembolic risk^61–63^. Our results indicate an increase in thrombin generation in breast cancer cells treated with Tamoxifen. Notably both cells lines are implicated in the secretion of Platelet Activating Factor (PAF) under thrombin stimulation, which in addition to mediating platelet aggregation, in an autocrine manner also enhances tumour progression^64,65^; however, T47D cells appeared to induce a more hypercoagulatory environment than MCF7 cells. Tamoxifen-treated T47D cells induced later stages of platelet activation associated with a loss of CD62P IPA and persistence of CD63 IPA reflecting hypercoagulation in both PRP and WB, compared to MCF7 cells. During early platelet activation, platelets undergo morphological alterations extending pseudopodia and spreading the cell membrane, while simultaneously releasing granular content^51,66^. CD62P is released from platelet α-granules and exposed on the plasma membrane; as activation progresses it is lost^50,66^. CD63, which is involved in recruitment of other platelets is said to be released from δ granules and lysosomes in the later stages of activation^51,67^; however, our results show that CD63 is expressed coupled with morphological and biochemical signs of early activation. Both activation markers, in addition to growth factors and agonists can also be released from platelets as microparticles^68,69^, which we observed in ultrastructural analysis.

In Model 2, MCF7 cells induced a similar level of hypercoagulation in Tamoxifen-treated blood constituents as Model 1; however, T47D cells showed an even greater propensity to induce hypercoagulation, losing expression of both platelet activation markers possibly to their role in mediating platelet aggregation or shed as microparticles, and essentially ‘exhausting’ platelets^70^. Notably, in platelet-poor plasma samples, fibrinogen deposits were readily available, indicating that the available thrombin, as opposed to facilitating fibrin formation as in MCF7 co-culture, may have rather competitively and selectively bound to PAR receptors which are reportedly more highly expressed on T47D cells than MCF7 cells^71,72^.

To better understand why MCF7 and T47D cells induce hypercoagulation variably, we assessed their sub-phenotype using markers that have clinical relevance. In both cell lines ERα expression was typically higher than ERβ expression, as expected. While the genomic actions of ERα are relatively well understood and the non-genomic actions of ERα less so^60^, there remains considerable lack of clarity on the role of ERβ in breast tumour progression. High ERβ expression has been implicated in breast tumour development, and notably in Tamoxifen-resistance^60^; however, more studies indicate that it may be associated with a more favourable response to treatment^73,74^, and that its reduction is associated with the transition to a more invasive phenotype^60^. Interestingly, T47D cells which are regarded as more invasive than MCF7 cells by expressing more proteins associated with tumorigenesis, growth and prevention of apoptosis^75^; presented with higher standard ERβ expression than MCF7 cells. Hormone-dependent tumours are identified by ERα overexpression, and while Tamoxifen mediates ER activity, downregulation of the receptor is associated with the transition to a more aggressive phenotype that does not respond to therapy, a common evasive strategy employed by tumour cells^60^. We identified that in both cell lines, exposure to WB resulted in a significant increase in ERβ expression; while in T47D cells, translocation of ERα to the nucleus may reflect engagement of genomic activities^60^. Recall that for this study, blood was collected from volunteers between days 1 and 10 of the menstrual cycle due to the lower levels of oestrogen and progesterone present^76^, with the aim of limiting the action of these hormones. Nevertheless, in both cell lines, and both models there was considerable variation induced by blood components; while MCF7 cells responded differentially, T47D cells demonstrated more of a pattern dominated by heightened ERβ. Furthermore, in Model 2 T47D cells specifically showed a reduction in ERα expression when compared to Model 1, indicating the efficacy of circulating Tamoxifen to not only heighten ERβ but downregulate ERα for a positive outcome. While WB better replicates the concept of circulatory interaction with tumour cells, many studies employ PRP to ascertain specific effects induced by platelets. However, we show that ER expression is sensitive to such conditions and postulate that the variation seen in our study is due to the generation of PRP, which even with soft centrifugation, provides a more concentrated source of growth factors^77^. This is notwithstanding that platelets themselves express ER^78,79^, possibly explaining the ‘exhaustion’ seen under direct Tamoxifen treatment; even PPP contains high concentrations of microparticles which contain a further host of growth factors, albeit at lesser levels^80^.

ER positive breast tumours are associated with tailored therapeutic approaches^81,82^, which increase the risk for thromboembolic complications. Our results highlight the procoagulant nature of breast cancer cells themselves and substantiate clinical studies indicating that Tamoxifen, regardless of model used, induces a hypercoagulatory environment, mediated by platelet interaction with tumour cells. The crucial role that platelets play in the tumour microenvironment is thus mediated by a host of secreted factors enabling cross-talk with tumour cells. Notably, changes in cytosolic and membrane ERα is postulated to relate to alterations of the metastatic potential of breast cancer cells, in which prothrombotic cytokines and thrombin^83^ are released to induce a hypercoagulable state to facilitate tumour progression^21,29,68,84^. This relationship is highlighted by the weak, yet positive correlation we identified between ERs, and the CD62P and CD63 IPA; indicating an association between tumour invasion profiles and hypercoagulation, however, it is noted that in addition to ER expression, other yet unknown factors may play a predictive role in defining hypercoagulation.

## Materials and methods

Human Research Ethics Clearance was obtained from the Human Ethics Research Committee (Medical), University of the Witwatersrand, Clearance Certificate Number M160826. Informed consent was obtained from all participants and all research was performed in accordance with the ethical guidelines.

### Cell culture

MCF7 cells and T47D cells obtained from the American Type Culture Collection (ATCC) were cultured as follows: MCF7 cells (P36) in DMEM (Dulbecco’s Modified Eagles Medium), with 10% FBS (Foetal Bovine Serum), 0.1% P/S (Penicillin/Streptomycin); T47D cells (P29) in RPMI (Roswell Park Memorial Institute Medium) with 0.2 U/mL bovine insulin, 10% FBS and 0.1% P/S. MCF10A, a non-tumorigenic breast epithelial cell line, kindly donated by Prof Raquel Duarte was propagated in MEGM (Mammary epithelial cell growth medium BulletKit), supplemented with 100 ng/mL cholera toxin (Merck, Johannesburg, South Africa; C8052).

For experimentation, cells were seeded at 1 × 10^5^ cells onto glass coverslips in a 24-well plate. MCF10A and MCF7 cells were seeded directly onto the glass whereas T47D cells were seeded onto glass coverslips coated with 5 mg/mL poly-d-lysine (Sigma-Aldrich; P6407) to facilitate attachment.

### Whole blood acquisition

Peripheral whole blood (WB) was obtained from healthy female donors (n = 6) between 19 and 30 years old into 3.2% sodium citrate vacuette coagulation tubes. The first 2 mL of blood drawn was discarded to exclude the effect of mechanically activated platelets^85^. Participant exclusion criteria included pregnancy, contraceptive use, previously identified autoimmune diseases or immunodeficiency, previous history of cancer, cancer, smoking, and consumption of anti-platelet and/or anti-coagulation medication in the previous 72 h. Blood was collected between days 1 and 10 of the menstrual cycle due to the lower levels of oestrogen and progesterone present within the circulating blood^76^.

### Establishment of Model 1 and Model 2 co-culture systems

For Model 1: Samples of WB were centrifuged at 200×*g* to yield platelet-rich plasma (PRP), and at 400×*g* to yield platelet-poor plasma (PPP). MCF7 and T47D cells were pre-treated with 2 μM Tamoxifen^30^ for 24 h prior to exposure to 200 μL WB, PRP or PPP for 2.5 min at room temperature (RT).

For Model 2: First WB was treated with 2 μM Tamoxifen for 1 h at RT. Subsequently, WB was centrifuged to yield PRP and PPP. Untreated MCF7 and T47D cells were then incubated with 200 µL Tamoxifen-treated WB, PRP or PPP for 2.5 min^50^ at RT (Supplementary Fig. S1).

Appropriate controls including diluent [0.1% DMSO (Dimethyl Sulfoxide)], untreated blood constituents and untreated cells were prepared for all experiments. Positive controls for accurate assessment of platelet activation were prepared by incubating lysed WB or PRP with 0.1 U/mL of human thrombin-α (SANBS, South Africa), a platelet agonist, for 5 min^50^.

### Sample preparation for thrombin activity assay

The release of thrombin was tested using a Thrombin Activity Assay Kit (Abcam; ab 197006). Following co-culture, blood samples or supernatant as required were aspirated, snap-frozen in liquid nitrogen and kept at − 80 °C. For the assay, samples were thawed on ice and diluted at 1/10 with assay buffer. 6 standards were prepared to achieve thrombin concentrations of; 0 ng/well, 5 ng/well, 10 ng/well, 15 ng/well, 20 ng/well, 25 ng/well. Diluted samples were added to their respective wells at 50 µL. Both standard and sample wells were incubated with thrombin substrate diluted in assay buffer. Fluorescence was measured at Ex/Em = 350/450 using a GloMax Promega Plate Reader in a kinetic mode every 2 min for 60 min at 37 °C, Department of Molecular Medicine and Haematology, University of the Witwatersrand.

### Sample preparation for flow cytometry

Lysed WB and PRP samples were resuspended in 150 μL Tyrode’s buffer. Samples were labelled with APC-conjugated mouse anti-human CD41a (BD Pharmingen; 559777), FITC-conjugated mouse anti-human CD62P (BD Pharmingen; 555523) and PE-Cy7 mouse anti-human CD63 (BD Pharmingen; 353010) at a concentration of 1:20 as determined using titration. Following compensation, experiments were conducted on an LSR Fortessa Flow Cytometer (BD Biosciences, South Africa). Events were recorded at 100,000 events per sample using FACSDiva Software (version 6.2) (BD Biosciences)^86^. The platelet population was initially gated using a forward scatter (FSC) voltage of 300 V and a side scatter (SSC) voltage of 275 V followed by gating a singlet population using FSC-height and FSC-area. The parent platelet population was identified and further gated based on expression of the CD41a (APC, 565 V). CD62P (FITC, 469 V) and CD63 (PE-CY7, 645 V). Interval gates^30,50^ were drawn to classify a graded level of each platelet activation marker determined using Geometric Mean Fluorescence Intensity (gMFI) of the platelet population (Fig. 2). Data acquired was exported into Microsoft Excel.

### Sample preparation for scanning electron microscopy

For both Models, WB samples underwent erythrocyte lysis using ammonium chloride buffer for 10 min at RT. Samples were centrifuged at 200×*g* for 5 min and the supernatant discarded. The pellet was resuspended in 150 μL Tyrode’s buffer. Thereafter, 20 μL each of; lysed WB, PRP or PPP were placed on glass coverslips in 24 well plates and incubated at 37 °C in 5% CO_2_ for 5 min for adhesion. Samples were washed in 0.1 M PBS on a microplate shaker for 20 min and fixed in 2.5% formaldehyde/glutaraldehyde for 15 min followed by rinsing thrice with 0.1 M PBS and secondary fixation in 1% osmium tetroxide. Following further rinsing in PBS, cells were dehydrated through an increasing series of ethanol (30%, 50%, 70%, 90%, and three times absolute ethanol), and dried using hexamethyldisilazane (HMDS). Samples were mounted onto aluminium stubs, coated by carbon evaporation and qualitative assessed using a FEI Nova 600 Scanning Electron Microscope with acceleration voltage set at 30 kV.

### Sample preparation for immunolocalisation of ERα and ERβ protein expression in breast cancer cells

MCF7 and T47D cells were washed thrice with 1 M PBS, fixed in 4% paraformaldehyde for 10 min at RT and washed with 0.1% Tween20/PBS. Cells were then incubated with 0.2% Triton X-100/PBS for 10 min at RT, washed three times with 0.1% Tween20/PBS for 5 min each and incubated in 1% BSA (Bovine Serum Albumin) in 0.1% Tween20/PBS for 30 min at RT. Cells were then incubated with Goat Anti-ERβ (Abcam, ab288) and Rabbit Anti-oestrogen Receptor ERα (Abcam, ab32063) primary antibodies, at concentrations 1:400 and 1:200 in 0.1% Tween20/PBS, respectively (concentrations were determined by conducting a dilution assay), at 4 °C overnight. Cells were then washed thrice with 0.1% Tween20/PBS and incubated with secondary antibodies Alexa Fluor 488 anti-rabbit (Life Technologies, Johannesburg, South Africa, A11008) and Alexa Fluor 594 anti-goat (Life Technologies, A11005), at 1:1000 respectively, for 2 h at RT in the dark. Cells were again washed thrice with 0.1% Tween20/PBS and nuclei were counterstained with DAPI. After a final wash with 0.1% Tween20/PBS coverslips were mounted onto slides using Fluoromount (Sigma-Aldrich, F4680). Standard immunocytochemical technical controls were included.

Photomicrographs were obtained using an Olympus iX51 Inverted Fluorescent Microscope with cellSens Dimension Software (version 1.11)^87^, School of Anatomical Sciences, University of the Witwatersrand. Photomicrographs were taken using filters: U-MNU2 to detect fluorescence from DAPI staining, U-MWIBA3 to detect cells stained with and expressing (ERα) Alexa Fluor 488 and U-MWIY2 to detect cells stained with and expressing (ERβ) Alexa Fluor 594. Photomicrographs were obtained in grayscale at 40× magnification and quantitatively analysed using CellProfiler software (version 3.1.5)^88^. In brief, DAPI photomicrographs were first processed to identify nuclei between a range between 40 and 100 pixels in diameter. Pixels that did not fall in this range or were on the boundary were discarded. The Background method was used for intensity thresholding and weighted variance was minimized with a non-adjusted “Threshold correction factor” indicated as 1. The lower bound was indicated at 0 and the upper bound was indicated at 1. Objects that were clumped were distinguished and separated using the “Intensity” method. Thereafter photomicrographs for Alexa Fluor 488 (ERα) were processed as ‘primary objects’. The Background method was used for intensity thresholding, again and weighted variance was minimized with a “Threshold correction factor” of 0.99. This was followed similarly, by identification of Alexa Fluor 594 (ERβ) in the corresponding photomicrograph. Images were further processed to obtain data on colocalization, cell size, shape and mean intensity of ERα and ERβ and exported to Microsoft Excel.

### Data analysis

For flow cytometry, the index of platelet activation (IPA) was calculated for Q2 or each interval (I1-5) such that:

$${\text{IPA}}^{{{\text{CD62P}}}} = {\text{gMFI}}\left( {{\text{CD62P}}^{ + } } \right){\text{X n}}\left( {{\text{CD41}}^{ + } {\text{CD62P}}^{ + } {\text{events}}} \right){\text{ or IPA}}^{{{\text{CD63}}}} = {\text{gMFI}}\left( {{\text{CD63}}^{ + } } \right){\text{X n}}\left( {{\text{CD41}}^{ + } {\text{CD63}}^{ + } {\text{events}}} \right)$$

All data from Microsoft Excel was then exported into Statistica Version 23 for analysis.

A Shapiro–Wilk test of normality determined that the data was not normally distributed. Moreover, to account for the limited sample size, non-parametric tests were used. The non-parametric Friedman test followed by a post-hoc Wilcoxon Matched Paired test, based on the dependency of blood constituents, was conducted to determine whether IPA, or thrombin availability or generation, or ER expression altered in response to hormone-therapy and blood constituent co-incubation. All tests were conducted at a confidence interval of 95%, and a significance level of 0.05.

## Supplementary information

Supplementary Information.

## Data Availability

The Authors will make datasets available on request subject to requirements from the Human Research Ethics Committee, University of the Witwatersrand.
