# Supplementary material for: Tamoxifen induces hypercoagulation and alterations in ERα and ERβ dependent on breast cancer sub-phenotype ex vivo
Source: Sci Rep. 2020 Nov 6;10:19256. doi: 10.1038/s41598-020-75779-y (PMC7648622; doi:10.1038/s41598-020-75779-y)
Supplement: Supplementary file 1 — Supplementary Information. [file 41598_2020_75779_MOESM1_ESM.docx]

Supplementary Figures - Changes

Tamoxifen induces hypercoagulation and alterations in ERα and ERβ dependent on breast cancer sub-phenotype *ex vivo*.

Pather, K. ^1*^, Augustine, T.N. ^1*^

^1^School of Anatomical Sciences, Faculty of Health Sciences, University of the Witwatersrand, 7 York Road, Parktown, 2193, Johannesburg, South Africa.

*Corresponding authors: Tanya N. Augustine ([tanya.augustine@wits.ac.za](mailto:tanya.augustine@wits.ac.za))/ Kyrtania Pather ([kyrtania.pather@wits.ac.za](mailto:kyrtania.pather@wits.ac.za))

Changes have been made to Accepted Supplementary Tables 1 and 2. These changes include:

- Bolding of “6,24E+05” and “6,26E+05” in Supplementary Table 1 to indicate significance
- Change of “*” to “#” in Supplementary Table 2 to indicate significance compared to M/T MEDPRP, this was altered to ensure the same symbol between Table 1 in-text and Supplementary Table 2 is the same, ensuring continuity.
- Change of “bold” to “underline” in Supplementary Table 2 to indicate significance compared to PRP, this was altered to ensure the same symbol between Table 1 in-text and Supplementary Table 2 is the same, ensuring continuity.


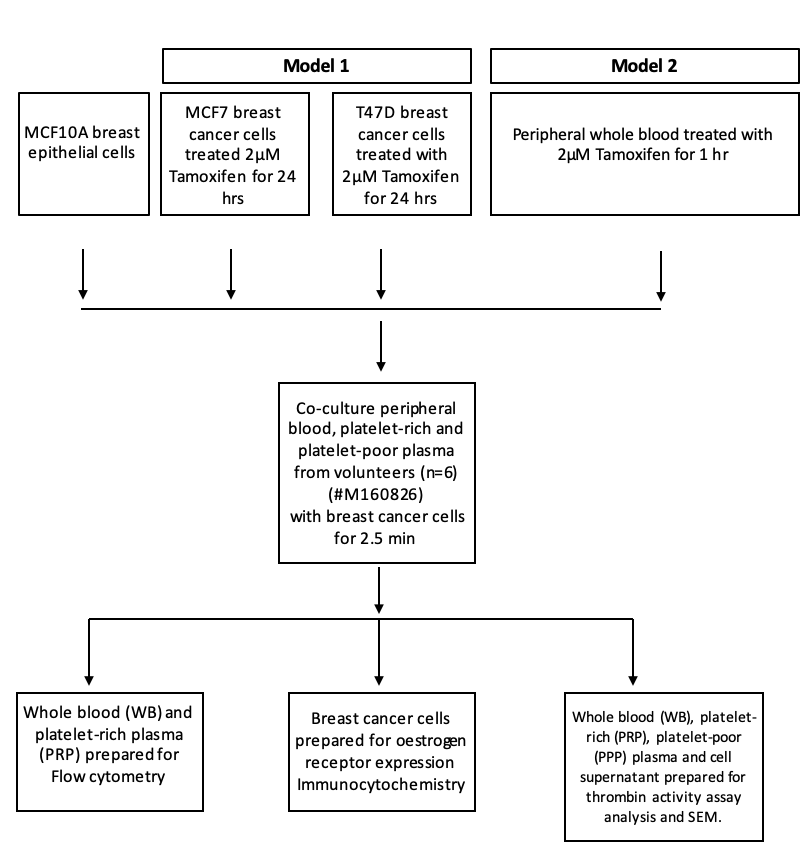


*Supplementary Figure 1:* ***Flow Diagram showing generation of Model 1 and Model 2, and subsequent assays.***

Supplementary Table 1: **Index of platelet activation markers CD62P and CD63, as well platelet progression (CD62P/CD63) of Tamoxifen-treatment of MCF7 and T47D cells exposed to WB (Model 1) and Tamoxifen treatment of WB exposed to MCF7 and T47D cells (Model 2).** Overall levels (Q2) and per interval gate levels; CD62P^low^ and CD63^low^ (I1 and I2), CD62P^medium^ and CD63^medium^ (I3), CD62P^high^ and CD63^high^ (I4 and I5) were assessed. Bold indicates samples which were significantly (p<0.05) different to untreated WB and (*) indicates samples significantly different to matched, WB exposed to T47D cells. Key: WB – untreated whole blood; WBThr: positive control, whole blood incubated with 0.1 U/ml of thrombin. WBTam: Tamoxifen-treated WB. MMEDWB: WB exposed to MCF7 cells. MDILWB: WB exposed to MCF7 cells treated with 0.1% DMSO, denoting the diluent control. MTAMWB: WB exposed to Tamoxifen-treated MCF7. WBTAMM: Tamoxifen-treated WB exposed to MCF7. TMEDWB: WB exposed to T47D cells. TDILWB: WB exposed to T47D cells treated with 0.1% DMSO, denoting the diluent control. TTAMWB: WB exposed to Tamoxifen-treated T47D. WBTAMT: Tamoxifen-treated WB exposed to T47D. Bold indicates samples which were significantly (p<0.05) different to untreated WB and (*) indicates samples significantly different to matched media cell lines (MMEDWB or TMEDWB). Italics refers to samples significantly different between cell lines.

| **Marker of Activation** | **Controls** | | | | | | | **Model 1** | | **Model 2** | |  |
| --- | --- | --- | --- | --- | --- | --- | --- | --- | --- | --- | --- | --- |
|  | **WB** | **WBThr** | **WBTam** | **MMEDWB** | **TMEDWB** | **MDILWB** | **TDILWB** | **MTAMWB** | **TTAMWB** | **WBTAMM** | **WBTAMT** |  |
| **Q2** | | | | | | | | | | | | |
| CD62P | 3,93E+05 | 4,99E+05 | 4,93E+05 | **6,33E+05** | 4,31E+05 | 5,48E+05 | 3,63E+05 | **6,24E+05** | **7,78E+05*** | **6,26E+05** | **6,01E+05*** |  |
| CD63 | 2,92E+06 | **3,35E+06** | 3,01E+06 | 3,65E+06 | **4,08E+06** | 3,15E+06 | 2,80E+06 | 3,07E+06 | **4,87E+06** | 3,64E+06 | **3,69E+06** |  |
| Platelet Progression (CD62P/CD63) | 1,35E-01 | 1,49E-01 | 1,64E-01 | 1,74E-01 | 1,06E-01 | 1,74E-01 | 1,30E-01 | 2,03E-01 | 1,60E-01 | 1,72E-01 | 1,63E-01 |  |
| **I1** | | | | | | | | | | | | |
| CD62P | 5,71E+04 | 4,78E+04 | 9,85E+04 | 1,67E+05 | 1,02E+05 | 1,19E+05 | 1,16E+05 | 1,55E+05 | **1,76E+05** | 1,30E+05* | **2,17E+05*** |  |
| CD63 | 1,78E+05 | **2,80E+05** | **5,63E+05** | **6,59E+05** | **4,53E+05** | 4,58E+05 | **4,38E+05** | **5,07E+05** | **6,66E+05*** | **5,70E+05** | **6,62E+05*** |  |
| **I2** | | | | | | | | | | | | |
| CD62P | 5,71E+04 | 2,28E+05 | 2,64E+05 | 3,41E+05 | 1,74E+05 | 2,93E+05 | 1,73E+05 | 3,58E+05 | **3,71E+05*** | 3,26E+05 | **3,71E+05*** |  |
| CD63 | 7,23E+05 | **1,07E+06** | **1,57E+06** | **1,70E+06** | **1,17E+06** | **1,09E+06** | **1,01E+06** | **1,64E+06** | **1,57E+06** | **1,64E+06** | **1,48E+06** |  |
| **I3** | | | | | | | | | | | | |
| CD62P | 1,69E+05 | 1,87E+05 | 1,27E+05 | **2,40E+05** | 2,23E+05 | **2,15E+05** | 1,44E+05 | 1,80E+05 | 2,57E+05 | **2,14E+05** | **1,95E+05** |  |
| CD63 | 1,22E+06 | **2,40E+06** | 1,28E+06 | 2,32E+06 | 2,02E+06 | 1,80E+06 | 1,24E+06 | 1,40E+06* | **2,76E+06** | 1,84E+06 | **1,99E+06** |  |
| **I4** | | | | | | | | | | | | |
| CD62P | 3,52E+04 | 2,30E+05 | **9,43E+03** | 3,59E+04 | **5,79E+04** | 4,59E+04 | 1,97E+04 | 3,02E+04 | **6,49E+03*** | **1,48E+04*** | **7,09E+03*** |  |
| CD63 | 1,07E+06 | 1,22E+06 | 3,92E+05 | 7,76E+05 | 1,25E+06 | **6,61E+05** | 5,58E+05 | 7,46E+05 | 2,82E+05* | **3,43E+05*** | **3,98E+05*** |  |
| **I5** | | | | | | | | | | | | |
| CD62P | 0,00E+00 | 0,00E+00 | 0,00E+00 | 0,00E+00 | 0,00E+00 | 0,00E+00 | 0,00E+00 | 0,00E+00 | 0,00E+00 | 0,00E+00 | 0,00E+00 |  |

Supplementary Table 2: **Index of platelet activation markers CD62P and CD63, as well platelet progression (CD62P/CD63) of Tamoxifen treatment of MCF7 and T47D cells exposed to PRP (Model 1) and Tamoxifen treatment of PRP exposed to MCF7 and T47D cells (Model 2).** Overall levels (Q2) and per interval gate levels; CD62P^low^ and CD63^low^ (I1 and I2), CD62P^medium^ and CD63^medium^ (I3), CD62P^high^ and CD63^high^ (I4 and I5) were assessed. Bold indicates samples which were significantly (p<0.05) different to untreated WB and (*) indicates samples significantly different to matched, WB exposed to T47D cells. Key: PRP – untreated Platelet-rich plasma; PRPThr: positive control, platelet-rich plasma incubated with 0.1 U/ml of thrombin. PRPTam – Tamoxifen-treated PRP. MMEDPRP: PRP exposed to MCF7 cells. MDILPRP: PRP exposed to MCF7 cells treated with 0.1% DMSO, denoting the diluent control. MTAMPRP: PRP exposed to Tamoxifen-treated MCF7. PRPTAMM: Tamoxifen-treated PRP exposed to MCF7. TMEDPRP: PRP exposed to T47D cells. TDILPRP: PRP exposed to T47D cells treated with 0.1% DMSO, denoting the diluent control. TTAMPRP: PRP exposed to Tamoxifen-treated T47D. PRPTAMT: Tamoxifen-treated PRP exposed to T47D. Bold indicates samples which were significantly (p<0.05) different to untreated WB and (#) indicates samples significantly different to matched media cell lines (MMEDPRP or TMEDPRP). Italics refers to samples significantly different between cell lines. Underline indicates samples significantly different to untreated PRP.

| **Marker of Activation** | **Controls** | | | | | | | **Model 1** | | **Model 2** | |
| --- | --- | --- | --- | --- | --- | --- | --- | --- | --- | --- | --- |
|  | **PRP** | **PRPThr** | **PRPTam** | **MMEDPRP** | **TMEDPRP** | **MDILPRP** | **TDILPRP** | **MTAMPRP** | **TTAMPRP** | **PRPTAMM** | **PRPTAMT** |
| **Q2** | | | | | | | | | | | |
| CD62P | **1,35E+06#** | 7,60E+05# | 7,73E+05 | 1,20E+06 | 1,20E+06 | 7,07E+05 | *1,76E+06#* | 8,62E+05# | *2,08E+06#* | 7,16E+05# | *1,52E+06* |
| CD63 | **6,99E+06#** | 8,41E+06# | 9,04E+06# | 1,25E+07# | *8,39E+06#* | 7,93E+06 | *1,14E+07#* | 6,11E+06* | *1,23E+07#* | 7,92E+06# | *1,01E+07****#*** |
| Platelet Progression (CD62P/CD63) | 1,53E-01 | 9,04E-02# | 8,55E-02# | *9,62E-02#* | 1,43E-01 | 8,92E-02# | *1,54E-01* | 1,41E-01* | 1,69E-01 | 9,03E-02 | 1,50E-01 |
| **I1** | | | | | | | | | | | |
| CD62P | 3,20E+05 | 2,27E+05 | 5,60E+05 | 4,94E+05 | 4,14E+05 | 3,06E+05 | 4,46E+05 | 3,39E+05 | 4,75E+05 | 4,80E+05 | *8,39E+05#* |
| CD63 | 1,16E+06 | 1,10E+06 | 4,37E+06# | 1,47E+06 | 1,49E+06 | 9,75E+05# | 1,70E+06 | 7,80E+05# | 1,02E+06 | 2,59E+06 | *3,61E+06#* |
| **I2** | | | | | | | | | | | |
| CD62P | 4,49E+05 | 3,79E+05 | 2,60E+05# | 8,43E+05 | 8,82E+05 | 4,94E+05# | 1,24E+06 | 6,08E+05 | 1,49E+06# | 3,11E+05# | *7,52E+05* |
| CD63 | 4,62E+06 | 3,38E+06 | 4,53E+06 | 8,34E+06 | 5,30E+06 | 5,32E+06 | 5,57E+06 | 4,02E+06# | 7,07E+06# | 4,59E+06 | 5,70E+06 |
| **I3** | | | | | | | | | | | |
| CD62P | 4,67E+04 | 1,66E+05 | 2,87E+04# | 1,77E+05 | 1,16E+05 | 4,68E+04 | 1,44E+05 | 6,05E+04 | 2,84E+05 | 1,93E+04# | 8,34E+04# |
| CD63 | 2,90E+06 | 4,65E+06 | 1,33E+06 | 5,59E+06 | 3,85E+06 | 4,26E+06 | 6,37E+06 | 2,39E+06 | 5,58E+06 | 1,63E+06# | 2,25E+06 |
| **I4** | | | | | | | | | | | |
| CD62P | 7,93E+03 | 9,29E+03 | 4,84E+03# | 4,91E+03 | 5,06E+03 | 0,00E+00 | 0,00E+00 | 3,07E+03 | 3,71E+03 | 0,00E+00 | 4,16E+03 |
| CD63 | 1,20E+05 | 1,02E+06# | 1,76E+05 | 2,39E+05 | 1,06E+05 | 1,47E+05 | 2,88E+05 | 1,22E+05 | 4,56E+05# | 1,04E+05 | 2,44E+05 |
| **I5** | | | | | | | | | | | |
| CD62P | 0,00E+00 | 0,00E+00 | 0,00E+00 | 0,00E+00 | 0,00E+00 | 0,00E+00 | 0,00E+00 | 0,00E+00 | 0,00E+00 | 0,00E+00 | 0,00E+00 |
| CD63 | 0,00E+00 | 3,57E+04 | 0,00E+00 | 4,02E+04 | 2,09E+04 | 0,00E+00 | 0,00E+00 | 0,00E+00 | 0,00E+00 | 0,00E+00 | 0,00E+00 |
